# Independent Risk Factors of Postoperative Coronal Imbalance after Adult Spinal Deformity Surgery

**DOI:** 10.3390/jcm12103559

**Published:** 2023-05-19

**Authors:** Alberto Ruffilli, Francesca Barile, Azzurra Paolucci, Marco Manzetti, Giovanni Viroli, Marco Ialuna, Fabio Vita, Tosca Cerasoli, Cesare Faldini

**Affiliations:** 1st Orthopaedic and Traumatologic Clinic, IRCCS Istituto Ortopedico Rizzoli, 40136 Bologna, Italy; alberto.ruffilli@ior.it (A.R.); francesca.barile@ior.it (F.B.); azzurra.paolucci@ior.it (A.P.); giovanni.viroli@ior.it (G.V.); marco.ialuna@ior.it (M.I.); fabio.vita@ior.it (F.V.); tosca.cerasoli@ior.it (T.C.); cesare.faldini@ior.it (C.F.)

**Keywords:** risk factors of coronal imbalance, inadequate correction, postoperative coronal imbalance, iatrogenic coronal imbalance, adult spinal deformity

## Abstract

The aim of the present study is to elucidate preoperative risk factors for inadequate correction of coronal imbalance and/or creation of new postoperative coronal imbalance (iatrogenic CIB) in patients who undergo surgery for Adult Spinal Deformity (ASD). A retrospective review of adults who underwent posterior spinal fusion (>5 levels) for ASD was performed. Patients were divided into groups according to the Nanjing classification: type A (CSVL < 3 cm), type B (CSVL > 3 cm and C7 plumb line shifted to major curve concavity), and type C (CSVL > 3 cm and C7 plumb line shifted to major curve convexity). They were also divided according to postoperative coronal balance in balanced (CB) vs. imbalanced (CIB) and according to iatrogenic coronal imbalance (iCIB). Preoperative, postoperative, and last follow-up radiographical parameters and intraoperative data were recorded. A multivariate analysis was performed to identify independent risk factors for CIB. A total of 127 patients were included (85 type A, 30 type B, 12 type C). They all underwent long (average levels fused 13.3 ± 2.7) all-posterior fusion. Type C patients were more at risk of developing postoperative CIB (*p* = 0.04). Multivariate regression analysis indicated L5 tilt angle as a preoperative risk factor for CIB (*p* = 0.007) and indicated L5 tilt angle and age as a preoperative independent risk factors for iatrogenic CIB (*p* = 0.01 and *p* = 0.008). Patients with a preoperative trunk shift towards the convexity of the main curve (type C) are more prone to postoperative CIB and leveling the L4 and L5 vertebrae is the key to achieve coronal alignment preventing the “takeoff phenomenon”.

## 1. Introduction

Maintaining or restoring global alignment is a fundamental goal of Adult Spinal Deformity (ASD) surgery [1,2,3]. Ideal balance after surgery produces favorable outcomes and avoids mechanical complications [4,5]. In fact, both sagittal and coronal imbalance (CIB) negatively influence functional outcomes and patient satisfaction [6,7,8]. However, while great attention has been devoted to sagittal imbalance [9,10], less has been paid to understanding the coronal plane. Recent literature indicates that greater postoperative CIB is associated with pain, loss of function, and decreased quality of life [11,12,13]. Moreover, adult patients suffering from CIB do not have many compensatory mechanisms due to the rigidity of the spine and frequent long fusions, even to the pelvis; therefore, immediate restoration of coronal balance (CB) is of the utmost importance. Recently, some authors have addressed the problem. Bao et al. [14] proposed a classification for coronal alignment and reported the preoperative direction of the trunk shift relative to the major curve to be a risk factor for persistent postoperative imbalance. This result was confirmed by other authors, who explained it with an inadequate correction of the lumbosacral fractional curve [5,15]. However, while some authors have analyzed the influence of the preoperative trunk shift on the postoperative balance [5,15], and others studied the L4 and L5 tilt [16,17,18], as far as the authors know, there is no study evaluating the correlation of all these preoperative variables (trunk shift, L4 tilt, L5 tilt, and sagittal parameters) on the postoperative balance, providing a comprehensive understanding of the preoperative radiographical risk factors for CIB. The aim of the present study was to elucidate preoperative risk factors for inadequate correction of coronal imbalance and/or creation of new postoperative coronal imbalance (iatrogenic CIB) in patients who undergo surgery for Adult Spinal Deformity (ASD).

## 2. Materials and Methods

### 2.1. Study Sample

After Institutional Review Board approval, a retrospective cohort study was conducted including consecutive patients who underwent posterior fusion for Adult Spinal Deformity (ASD) in our institution between 1 January 2012 and 1 March 2020. Inclusion criteria were age at surgery > 18 years; a diagnosis of Adult Spinal Deformity (ASD) Aebi types 1 or 2, with main curve Cobb angle > 30°; posterior fusion of at least 5 levels with all-screws constructs extended distally to L5 or below; available preoperative, postoperative and last follow-up X-rays; and minimum 2-year follow-up. Patients with congenital or neuromuscular scoliosis, prior spinal operations, no coronal deformity (main curve Cobb angle < 10°), ankylosing spondylitis, Parkinson disease or other neurological disorders, and spinal neoplasms were excluded. Basic demographic and surgical data were recorded. The following radiographical parameters were assessed preoperatively, postoperatively (just before discharge), and at the last follow-up on long-cassette standing X-rays of the entire spine: Cobb angle of the main curve and of the lumbosacral fractional curve (LSF), main curve apex, coronal vertical axis (CVA, distance between the C7 plumb line and the central sacral vertical line CSVL), sagittal vertical axis (SVA), lumbar lordosis (LL), thoracic Kyphosis (TK), and spinopelvic parameters (L1-L4 lordosis, L4-S1 lordosis, Pelvic Tilt PT, Pelvic Incidence PI, Sacral Slope SS, PI-LL). Coronal imbalance (CIB) was defined as a CVA > 30 mm. All the measures were taken with Surgimap Software (Surgimap Spine Software^®^, Version 2.0.6, New York, NY, USA) by two experienced surgeons. Postoperative complications (mechanical, systemic, and infective) and revisions were recorded up to the last follow-up. Patients were stratified in two groups (coronally balanced CB vs. coronally imbalanced CIB) based on absolute postoperative CVA. Then, the iatrogenic CIB (iCIB) subgroup of patients was identified: iatrogenic CIB was defined as a postoperative CVA > 30 mm in patients with a preoperative normal alignment (CVA < 30 mm). Iatrogenic CIB patients were compared with those whose CVA was normal both preoperatively and postoperatively. Moreover, according to the Nanjing classification [14], three groups of patients were identified: type A if CVA < 30 mm, type B if CVA > 30 mm and C7PL shifted to the concave side of the main curve and type C if CVA > 30 mm and C7PL shifted the convex side of the main curve.

### 2.2. Statical Analysis

Descriptive statistics were used to summarize patients’ demographic characteristics and radiographic data. Categorical data were presented as frequencies and percentages, while continuous data were presented as mean ± standard deviation (SD). The present study was divided into steps. First, all pre- and postoperative radiological parameters were analyzed with a paired t-test to compare the differences. Then, comparisons between the groups (coronally balanced vs. imbalanced patients and type A vs. B vs. C) were carried out by independent t-tests or Fisher exact test. Chi-squared tests were performed to analyze the relationship between pre- and postoperative coronal imbalance. Bonferroni post-hoc correction of significance levels was used when there were multiple comparisons. To calculate the measure of effect for continuous variables among three groups (preoperative types A, B and C9), Analysis of Variance (ANOVA) was used, with η^2^ as effect size measure. Finally, multivariate analysis was conducted to identify preoperative risk factors for postoperative imbalance and for iatrogenic imbalance. A *p*-value < 0.05 was considered significant. All analyses were performed with Jamovi software (the Jamovi project—jamovi Version 1.6.2021).

## 3. Results

### 3.1. Demographic Data

One hundred twenty-seven patients (116 females and 11 males, average age 56.3 ± 12.9 years) were included. Baseline demographics and surgical data are reported in Table 1.

Surgery consisted of a long (average levels fused 13.3 ± 2.7) all-posterior thoracolumbar fusion with high density pedicle screw constructs that terminated above T9 in 113/127 patients (88.9%) and in S1 or pelvis in 69/127 (54.3%) patients. All patients were operated on with the same technique; the fusion area was preoperatively defined according to the Lenke criteria; in the presence of disc degeneration at the level distal to the planned Lower Instrumented Vertebra (LIV), the fusion area was extended distally; when the LSF curve showed low flexibility (<20° Cobb at supine radiographs), the fusion area was extended to the pelvis. The mean number of posterior column osteotomies per patient was 6.02 ± 1.9; three column osteotomies were performed on five patients (3.9%). Posterior lumbar interbody fusion was performed on 84 patients (66.2%). Coronal, sagittal, and spinopelvic parameters improved after surgery (Table 2), and they did not significantly change between postoperative and last follow-up X-rays.

Preoperatively, most patients (85/127, 67%) had Type A coronal alignment, while 30 (23.6%) were shifted to the concavity of the main curve (type B) and 12 (9.4%) were shifted to the convexity (type C), Table 3.

Type C patients were more at risk of having a higher postoperative CVA (*p* = 0.04, η^2^ 0.3). Patients with type B coronal imbalance had a significantly more severe main curve (*p* = 0.02, η^2^ 0.07) and higher SVA (*p* < 0.001, η^2^ 0.3) and CVA (*p* < 0.001, η^2^ 0.6), while type A patients had higher LL (*p* < 0.001, η^2^ 0.2), L4-S1 LL (*p* = 0.005, η^2^ 0.3), and SS (*p* = 0.01, η^2^ 0.1) and lower PT (*p* = 0.04, η^2^ 0.09). As for L4-S1 Lumbosacral Fractional (LSF) curve and L4 and L5 tilt, there was no preoperative difference between the groups; however, a strong difference was recorded in postoperative L4 tilt (*p* = 0.005, η^2^ 0.2), with type C patients showing the highest value (15.7 ± 5.2).

### 3.2. Coronally Balanced (CB) vs. Imbalances (CIB) Patients

**Figure 1:**
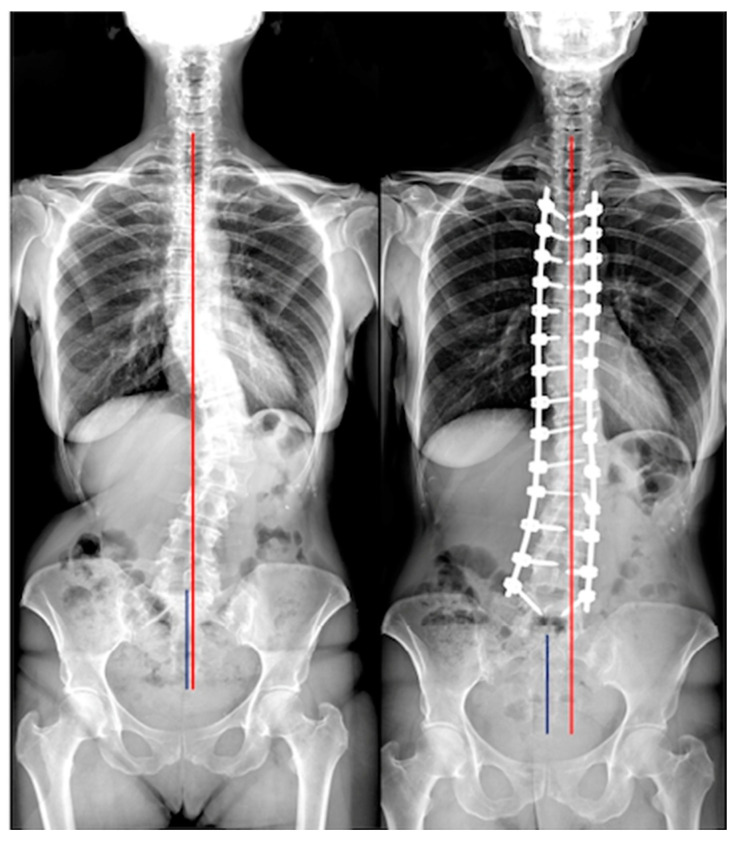
A case classified A both preoperative and postoperative. Red line: C7 plumb line; Blue line: central sacral vertical line.

**Figure 2:**
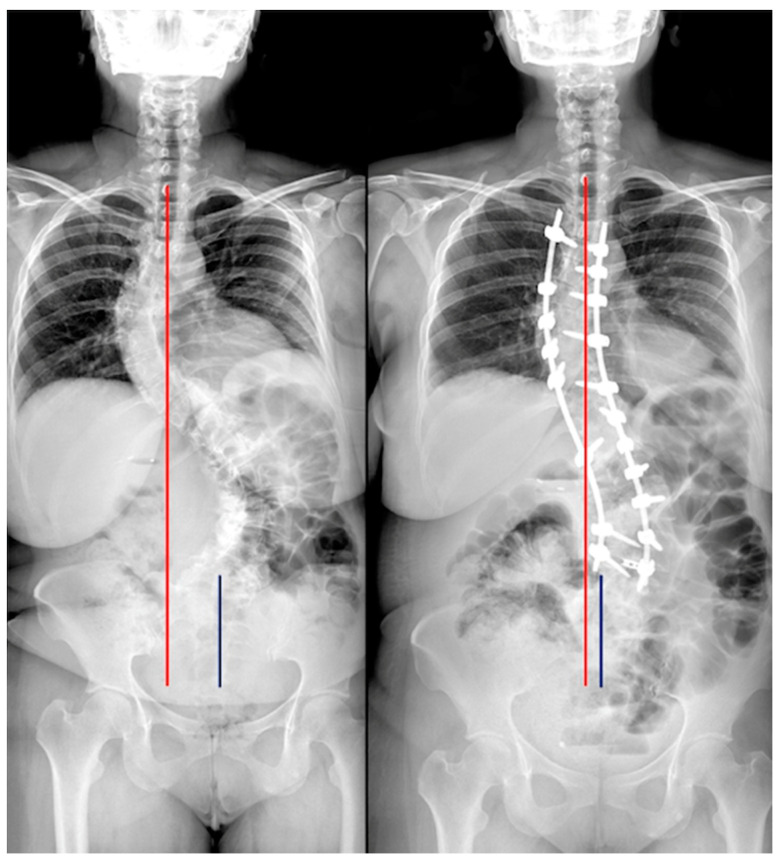
A case classified B preoperative and A postoperative. Red line: C7 plumb line; Blue line: central sacral vertical line.

**Figure 3:**
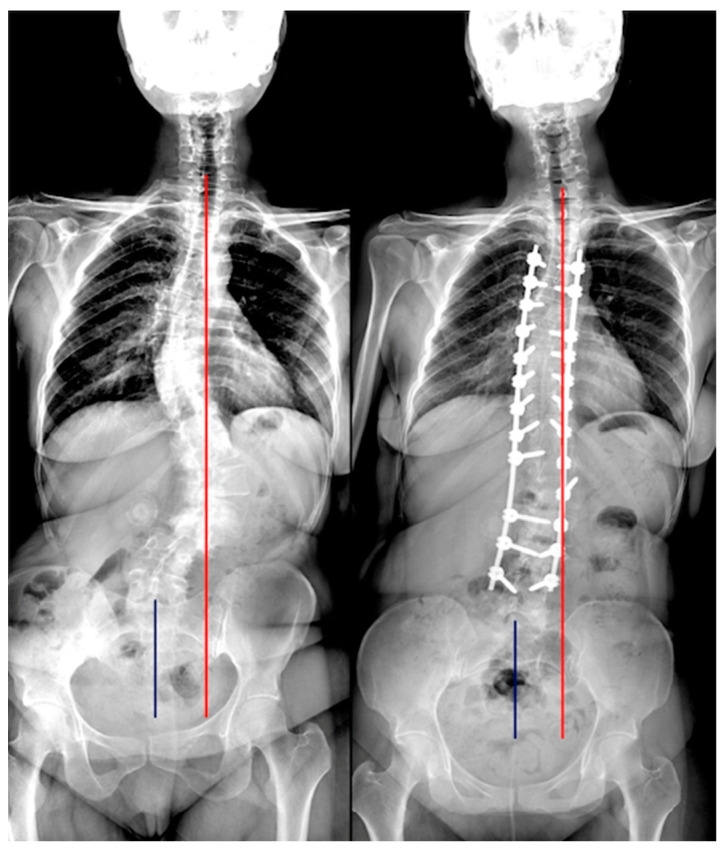
A case classified C both in preoperative and postoperative. Red line: C7 plumb line; Blue line: central sacral vertical line.

Of the 127 included patients, 84 (66.1%) had good postoperative coronal balance (CB), while 43 (33.9%) had coronal malalignment (CIB) (Figure 1, Figure 2 and Figure 3, Table 4).

Preoperatively, the average CVA and main curve Cobb angle were 25.2 ± 22.6 mm and 47.3 ± 17.4° in the CB group, and 30.7 ± 30.5 mm and 49.4 ± 15.9° in the CIB group (*p* > 0.05). The two groups were significantly different in preoperative L5 tilt (11.6 ± 7.6° vs. 15.1 ± 5.8°, *p* = 0.04). Multivariate regression analysis indicated L5 tilt angle as a preoperative risk factor for CIB (*p* = 0.007). Both groups showed significant improvements in all coronal and sagittal parameters after surgery, without significant differences. However, postoperative L4 and L5 tilts were significantly lower in the balanced group (*p* = 0.03 and *p* = 0.02, respectively). There was no difference in complications and revision rate between the two groups (*p* = 0.9 and *p* = 0.4).

### 3.3. Risk Factors for Iatrogenic Coronal Imbalance

Among the 85 patients with normal preoperative CVA (preoperative type A), 28 (33%) had iatrogenic CIB (Table 5).

The two groups were not different in preoperative CVA, SVA, major curve, and lumbosacral fractional curve Cobb; however, patients with iCIB showed a greater preoperative L5 tilt (14.9 ± 5.7° vs. 11.9 ± 7.5°, *p* = 0.04) and a greater postoperative L4 tilt (13.3 ± 5.5° vs. 9.3 ± 7.3°, *p* = 0.04). Multivariate regression analysis indicated L5 tilt angle and age as a preoperative independent risk factor for iatrogenic CIB (*p* = 0.01 and *p* = 0.008, respectively). Both groups showed improvements in all coronal and sagittal parameters after surgery, without significant differences. There was no difference in complications and revision rate between the two groups (*p* = 0.9 and *p* = 0.8).

## 4. Discussion

The aim of the present study was to elucidate preoperative risk factors for inadequate correction of coronal imbalance and/or creation of new postoperative coronal imbalance (iatrogenic CIB) in patients who undergo surgery for Adult Spinal Deformity (ASD). Our main findings are twofold: first, patients with a preoperative trunk shift towards the convexity of the main curve (type C) are at higher risk of postoperative CIB; second, preoperative L5 tilt is an independent risk factor for both coronal imbalance and iatrogenic coronal imbalance. In the present study, preoperatively balanced (type A) and imbalanced (type B and C) patients did not differ significantly on the coronal plane (lumbosacral fractional curve severity, L4, and L5 tilt). However, postoperatively, type C patients showed the highest postoperative CVA (*p* = 0.04, η^2^ 0.3) and the highest L4 tilt angle (15.7 ± 5.2, *p* = 0.005), while the major curve Cobb angle was similar between the groups: this might indicate that, in type C patients, correction of the major curve has been excessive and not tailored to match correction of the LSF curve, failing to restore a good balance [14,16,19]. Our result is in line with the existing literature [5,14]. An explanation for the high risk of CIB in patients with a preoperative type C imbalance can be found in the surgical strategy: in fact, traditional approaches aiming to correct coronal deformities consist of achieving distraction of the concave side and compression of the convex side (for example, by placing cages in concavity or by performing asymmetric tricolumn osteotomies); however, while these maneuvers can correct the coronal plane in type A and B patients, they may aggravate the inclination of the trunk in type Cs [14,16,17]. As for independent risk factors, the multivariate analysis demonstrated that preoperative L5 tilt is an independent risk factor for both postoperative coronal imbalance (CIB, *p* = 0.007) and iatrogenic imbalance (iCIB, *p* = 0.01). These results are in line with the findings of other authors [17,20,21]. Even if the direction of the tilt (consistent or opposite to the direction of the CVA) was not considered [17], a high value indicates that the preoperative imbalance is partly driven by the lumbar/lumbosacral compensatory curve and that the surgical strategy should aim at restoring a horizontal foundation, thus avoiding the takeoff phenomenon [14]. Regarding surgical strategies for preventing CIB, some suggestions were published by Bao et al. and Obeid et al. [22], sharing common concepts: in fact, both consider addressing the lumbosacral fractional curve of utmost importance and report that leveling the L5 coronal tilt is an appropriate intraoperative landmark for good balance. This is even more important when a fusion to the pelvis is planned: in fact, the chance of spontaneous correction of postoperative CIB is lower when the Lower Instrumented Vertebra (LIV) is below L5 [14,15]. Nevertheless, whether postoperative coronal malalignment had an impact on postoperative clinical outcomes appears to be less clear. In fact, no difference was seen in the rate of complications and revision surgery, and the existing literature reports mixed results. While Obeid et al. stated that the correction of CIB was an important factor for improving surgical outcomes, other authors concluded that the magnitude of coronal deformity and the extent of coronal correction are less critical parameters in adult scoliosis [23,24]. Therefore, the impact of postoperative CIB on patients remains a wide area of study. Moreover, our results show a significant improvement of CVA at last follow-up (*p* = 0.004). This finding, which is in line with Zuckerman et al. [5] makes it even more difficult to draw conclusions on the significance of postoperative coronal malalignment. The results of this study should be considered in the context of its limitations. First, data collection was subject to the limitations of retrospective data extraction from electronic records. Then, this was a single center and single surgeon study, and all surgeries were performed with all-posterior strategies; moreover, the vast majority of included patients were female; these considerations may represent biases and limit the generalizability of the results. Not employing anterior surgery in this cohort of patients may represent a selection bias that should not be ignored. Another potential limitation is the threshold of coronal balance; in fact, its definition in the literature ranges from 2 [17] to 4 cm [23]. In our study, 3 cm was chosen as a threshold because it is the most frequently used [4,5,16,17], in line with the Nanjing classification [14]. Moreover, measures were taken manually on simple anteroposterior and lateral radiographs: this may limit the reproducibility of the results, especially those about the spinopelvic parameters. Furthermore, we did not comment on clinical outcomes for the three subgroups (type A, B, and C) because the focus of the study was the assessment of preoperative radiographic risk factors for postoperative coronal imbalance; then, intraoperative and postoperative parameters (such as fusion levels) that may influence postoperative balance were not taken into account because they were not in line with the aim of the study. Finally, longer follow-up data are needed to enforce our results, because spontaneous resolution (or at least improvement) of CIB might be possible over time. Despite these limitations, this manuscript has a relatively large cohort and a long follow-up and reports new findings. Therefore, the authors believe it contributes to the knowledge on coronal alignment in ASD.

## 5. Conclusions

In conclusion, our results suggest that patients with a preoperative trunk shift towards the convexity of the main curve (type C imbalance) are more prone to postoperative CIB and that leveling the L4 and L5 vertebrae is the key to achieve coronal alignment, preventing the “takeoff phenomenon” [14]: this is extremely useful information. In fact, while determining intraoperative global coronal balance can be difficult, an intraoperative posteroanterior lumbar X-ray can easily be performed offering the necessary visualization of L4 and L5 tilt.

## Figures and Tables

**Table 1:** Patients’ characteristics. N: number; F: female; M: male; UIV: upper instrumented vertebra; LIV: lower instrumented vertebra; PLIF: posterior lumbar interbody fusion.

Characteristics	Values
N. of patients	127
Age at surgery (years)	56.3 ± 12.9
Gender (F:M)	116:11 (93%:7%)
Follow-up (months)	47.5 ± 12.2
Curve apex (mode)	L2
UIV (T9 or above: T10 or below)	113:14 (88.9%:11.1%)
LIV (pelvic and S1:L5 or above)	69:58 (54.3%:45.7%)
PLIF (yes:no)	84:43 (66.2%:33.8%)
Complications	27 (21.2%): 20 mechanical, 3 systemic, 4 infective
Revision surgery	27 (21.2%)

**Table 2:** Pre- and postoperative characteristics of the patients. CVA: coronal vertical axis; SVA: sagittal vertical axis; TK: thoracic Kyphosis (T4-T12); LL: lumbar lordosis; PI: pelvic incidence; PT: pelvic tilt; SS: sacral slope.

Parameter	Preoperative	Postoperative	*p* Value
CVA (mm)	51.2 ± 8.9	28.1 ± 15.5	***0.20***
Major curve Cobb (°)	51.9 ± 32.7	27.5 ± 12.9	***<0.001***
L4-S1 Cobb (°)	15.3 ± 3.4	6.5 ± 4.9	***<0.001***
L4 tilt (°)	20.8 ± 2.5	11.5 ± 5.1	***<0.001***
L5 tilt (°)	14.5 ± 5.7	12.0 ± 1.6	***<0.001***
SVA (mm)	33.5 ± 47.4	26 ± 14.3	***<0.001***
TK (°)	27.8 ± 11.1	20.4 ± 1.5	***0.1***
LL (°)	13.3 ± 18.8	38.2 ± 10.7	***<0.001***
LL L1-L4 (°)	2 ± 2.8	16.7 ± 3.9	***<0.001***
LL L4-S1 (°)	15.2 ± 21.6	30.2 ± 9.1	***0.007***
PI	27.5 ± 38.9	53.3 ± 17.2	***0.9***
PT	14.2 ± 20.1	25.4 ± 7.8	<0.001
SS	13.3 ± 18.8	27.8 ± 9.4	0.018
PI-LL	14.2 ± 20.1	15.5 ± 6.6	<0.001

**Table 3:** Differences between preoperative types A (balanced), B (CVA > 3 mm and trunk shifted towards concavity), and C (CVA > 3 mm and trunk shifted towards convexity). Preop: preoperative; N: number; CIB n.: number of coronal imbalanced patients; CVA: coronal vertical axis; SVA: sagittal vertical axis; TK: thoracic kyphosis; LL: lumbar lordosis; PI: pelvic incidence; PT: pelvic tilt; SS: sacral slope.

	Parameter	Preop Type A	Preop Type B	Preop Type C	*p* Value
	N	85 (67%)	30 (23.6%)	12 (9.4%)	-
	Age (years)	54.6 ± 12.8	60.3 ± 13.5	59.5 ± 9.9	0.09
	CIB n.	29 (34%)	13 (43.3%)	7 (58.3%)	-
	CVA (mm)	12.8 ± 8.2	57.4 ± 26.6	52.1 ± 17.9	**<0.001**
	Major curve Cobb (°)	48.6 ± 17.2	50.1 ± 17.1	38.4 ± 11.2	**0.02**
	L4-S1 Cobb (°)	14.6 ± 8.6	14.5 ± 11.4	20.8 ± 8.9	0.1
	L4 tilt (°)	18.1 ± 9.4	16.4 ± 7.6	23.5 ± 8.7	0.07
	L5 tilt (°)	12.8 ± 7.3	12.9 ± 7.5	11.7 ± 5.9	0.8
	SVA (mm)	46.0 ± 48.3	101 ± 67.1	85 ± 60.8	**<0.001**
Preoperative	TK (°)	28.0 ± 14.8	36.6 ± 60.3	18.0 ± 16.5	0.1
	LL (°)	42.9 ± 18.7	25.7 ± 21.0	27.0 ± 18.7	**<0.001**
	LL L1-L4 (°)	13.7 ± 9.7	13.6 ± 11.2	13.2 ± 10.4	0.9
	LL L4-S1 (°)	46.8 ± 16.9	35.0 ± 18.8	31.6 ± 20.9	**0.005**
	PI	53.4 ± 18.8	50.5 ± 26.7	52.7 ± 19.0	0.9
	PT	23.4 ± 12.0	30.2 ± 11.6	28.3 ± 15.2	**0.04**
	SS	32.2 ± 11.8	25.4 ± 10.8	24.4 ± 13.8	**0.01**
	PI-LL	21.0 ± 16.2	24.0 ± 21.3	29.7 ± 23.2	0.4
	CVA (mm)	22.3 ± 17.9	22.0 ± 19.7	38.0 ± 21.5	0.07
	Major curve Cobb (°)	20.9 ± 13.1	18.6 ± 14.1	20.5 ± 9.0	0.7
	L4-S1 Cobb (°)	7.8 ± 5.9	7.5 ± 7.6	12.0 ± 6.0	0.08
	L4 tilt (°)	10.1 ± 6.7	8.9 ± 7.8	15.7 ± 5.2	**0.005**
Postoperative	L5 tilt (°)	8.2 ± 4.9	7.4 ± 6.8	12.0 ± 6.0	0.1
	SVA (mm)	32.9 ± 30.1	24.9 ± 22.1	26.8 ± 17.7	0.3
	TK (°)	24.4 ± 9.8	25.3 ± 12.0	23.4 ± 12.4	0.9
	LL (°)	48.0 ± 15.8	43.6 ± 16.6	45.1 ± 15.1	0.4
	LL L1-L4 (°)	19.9 ± 11.3	16.8 ± 11.7	16.5 ± 8.4	0.3
	LL L4-S1 (°)	38.9 ± 12.7	36.1 ± 15.2	38.6 ± 14.6	0.7
	PI	53.9 ± 14.8	47.8 ± 19.7	50.9 ± 17.0	0.3
	PT	21.1 ± 11.3	21.1 ± 8.4	21.9 ± 8.0	0.9
	SS	33.4 ± 15.5	32.0 ± 8.2	33.5 ± 6.4	0.8
	PI-LL	12.0 ± 9.3	8.9 ± 6.9	8.3 ± 5.8	0.08
	Complications	18 (21.2%)	7 (23.3%)	2 (16.7)	0.3
	No	67	23	10	
	Mechanical	14	4	2	
	Systemic	3	0	0	
	Infective	1	3	0	
Revision surgery	Revision surgery	19 (22.3%)	6 (20%)	2(16.7%)	0.9

**Table 4:** Differences between postoperatively balanced (CB) and unbalanced (CIB) patients (CVA > 30 mm). N: number; CVA: coronal vertical axis; SVA: sagittal vertical axis; TK: thoracic kyphosis; LL: lumbar lordosis; PI: pelvic incidence; PT: pelvic tilt; SS: sacral slope.

	Parameter	CB	CIB (CVA > 30 mm)	*p* Value Univariate	Log Odds Multivariate	*p* Value Multivariate
	N.	84 (66.1%)	43 (33.9%)	-	-	-
	Age	55.8 ± 14.1	57.6 ± 10.0	0.3	−0.04	0.07
Preoperative	CVA (mm)	25.2 ± 22.6	30.7 ± 30.5	0.1	−0.01	0.17
	Major curve Cobb (°)	47.3 ± 17.4	49.4 ± 15.9	0.5	−0.01	0.36
	L4-S1 Cobb (°)	14.2 ± 8.7	17.2 ± 10.6	0.4	−0.05	0.13
	L4 tilt (°)	17.1 ± 9.5	20.3 ± 8.0	0.1	0.06	0.18
	L5 tilt (°)	11.6 ± 7.6	15.1 ± 5.8	**0.04**	−0.12	**0.007**
	SVA (mm)	59.2 ± 52.3	69.6 ± 70.9	0.3	−0.003	0.64
	TK (°)	31.0 ± 38.0	25.4 ± 15.1	0.4	0.03	0.29
	LL (°)	38.2 ± 21.0	35.7 ± 20.2	0.5	−0.02	0.58
	LL L1-L4 (°)	14.3 ± 10.7	12.2 ± 8.5	0.4	0.02	0.48
	LL L4-S1 (°)	42.0 ± 18.8	43.6 ± 18.6	0.7	−0.03	0.19
	PI	52.7 ± 18.4	52.5 ± 24.9	0.9	0.008	0.47
	PT	25.2 ± 11.8	26.0 ± 13.8	0.6	−0.02	0.39
	SS	29.8 ± 12.1	30.0 ± 12.5	0.9	−0.002	0.96
	PI-LL	22.9 ± 17.5	21.6 ± 19.7	0.8	0.006	0.61
Postoperative	CVA (mm)	12.7 ± 8.7	45.1 ± 13.2	**<0.001**		
	Major curve Cobb (°)	19.8 ± 12.1	21.4 ± 14.5	0.6		
	L4-S1 Cobb (°)	7.5 ± 6.6	9.2 ± 6.1	0.3		
	L4 tilt (°)	9.3 ± 7.4	12.5 ± 5.9	**0.03**		
	L5 tilt (°)	7.5 ± 5.5	10.1 ± 5.3	**0.02 **		
	SVA (mm)	33.2 ± 27.7	25.1 ± 26.6	0.1		
	TK (°)	25.4 ± 10.3	22.8 ± 11.0	0.2		
	LL (°)	46.5 ± 16.6	47.0 ± 14.7	0.9		
	LL L1-L4 (°)	19.3 ± 11.8	18.0 ± 10.0	0.6		
	LL L4-S1 (°)	37.7 ± 13.5	39.1 ± 13.5	0.6		
	PI	51.7 ± 16.6	53.2 ± 16.1	0.6		
	PT	20.7 ± 10.2	22.1 ± 10.9	0.4		
	SS	32.9 ± 12.4	33.5 ± 15.7	0.2		
	PI-LL	11.0 ± 8.7	10.8 ± 8.5	0.9		
	Complications	19 (22.3%)	8 (19%)	0.9		
	No	66	34			
	Mechanical	14	6			
	Systemic	2	1			
	Infective	3	1			
Revision surgery		20 (23.5%)	7 (16.7%)	0.4		

**Table 5:** Differences between patients whose CVA improved (or remained stable and <30 mm) after surgery (CB) and patients whose CVA worsened (iatrogenic CIB, iCIB). N: number; CVA: coronal vertical axis; SVA: sagittal vertical axis; TK: thoracic kyphosis; LL: lumbar lordosis; PI: pelvic incidence; PT: pelvic tilt; SS: sacral slope.

	Parameter	CB	iCIB (CVA Postop > CVA Preop and >30 mm)	*p* Value	Log Odds Multivariate	*p* Value Multivariate
	N.	92 (72.4%)	35 (27.6%)	-	-	-
	Age	55.6 ± 13.6	58.5 ± 10.7	0.3	0.07	**0.008**
Preoperative	CVA (mm)	29.7 ± 27.5	20.1 ± 18.0	0.06	−0.02	0.07
	Major curve Cobb (°)	47.9 ± 17.1	48.2 ± 16.4	0.9	0.009	0.56
	L4-S1 Cobb (°)	14.7 ± 10.1	16.4 ± 7.4	0.4	0.02	0.66
	L4 tilt (°)	17.2 ± 9.6	20.7 ± 7.1	0.06	0.02	0.65
	L5 tilt (°)	11.9 ± 7.5	14.9 ± 5.7	**0.04**	0.07	**0.01**
	SVA (mm)	65.7 ± 58.6	54.9 ± 60.1	0.4	0.003	0.66
	TK (°)	31.0 ± 36.8	24.1 ± 12.7	0.3	−0.05	0.12
	LL (°)	37.0 ± 21.6	38.2 ± 18.3	0.8	0.05	0.22
	LL L1-L4 (°)	14.1 ± 10.6	12.3 ± 8.5	0.4	−0.02	0.33
	LL L4-S1 (°)	42.5 ± 18.5	42.6 ± 19.2	0.9	0.07	0.99
	PI	53.5 ± 18.2	50.4 ± 26.4	0.5	−0.01	0.37
	PT	25.7 ± 12.2	24.7 ± 13.2	0.7	0.008	0.76
	SS	29.8 ± 12.0	29.9 ± 12.7	0.9	−0.01	0.78
	PI-LL	22.9 ± 18	20.9 ± 19.0	0.6	−0.007	0.63
Postoperative	CVA (mm)	16.4 ± 13.8	42.7 ± 16.0	**<0.001**		
	Major curve Cobb (°)	20.4 ± 12.6	20.1 ± 14.0	0.8		
	L4-S1 Cobb (°)	7.7 ± 6.7	9.2 ± 5.8	0.2		
	L4 tilt (°)	9.3 ± 7.3	13.3 ± 5.5	**0.04**		
	L5 tilt (°)	8.1 ± 5.8	9.3 ± 4.8	0.3		
	SVA (mm)	32.1 ± 27.2	26.0 ± 28.1	0.3		
	TK (°)	25.6 ± 10.6	21.6 ± 10.1	0.06		
	LL (°)	46.7 ± 15.9	46.5 ± 16.1	0.9		
	LL L1-L4 (°)	18.5 ± 11.5	19.9 ± 10.5	0.5		
	LL L4-S1 (°)	7.7 ± 6.7	9.2 ± 5.8	0.3		
	PI	51.8 ± 16.0	53.2 ± 17.4	0.7		
	PT	20.1 ± 9.9	23.8 ± 11.4	0.08		
	SS	32.4 ± 14.4	34.9 ± 11.0	0.4		
	PI-LL	10.8 ± 8.6	11.3 ± 8.8	0.7		
	**Complications**	19 (20.6%)	8 (22.8%)	0.9		
	No	73	27			
	Mechanical	14	6			
	Systemic	2	1			
	Infective	3	1			
Revision surgery		19 (20.6%)	8 (22.8%)	0.8		

## Data Availability

Not applicable.

## References

[1] Aebi M. (2005). The Adult Scoliosis. Eur. Spine J..

[2] Bradford D.S., Tay B.K., Hu S.S. (1999). Adult Scoliosis: Surgical Indications, Operative Management, Complications, and Outcomes. Spine.

[3] Oskouian R.J.J., Shaffrey C.I. (2006). Degenerative Lumbar Scoliosis. Neurosurg. Clin..

[4] Bao H., Liu Z., Zhang Y., Sun X., Jiang J., Qian B., Mao S., Qiu Y., Zhu Z. (2019). Sequential Correction Technique to Avoid Postoperative Global Coronal Decompensation in Rigid Adult Spinal Deformity: A Technical Note and Preliminary Results. Eur. Spine J..

[5] Zuckerman S.L., Lai C.S., Shen Y., Lee N.J., Kerolus M.G., Ha A.S., Buchanan I.A., Leung E., Cerpa M., Lehman R.A. (2021). Incidence and Risk Factors of Iatrogenic Coronal Malalignment after Adult Spinal Deformity Surgery: A Single-Center Experience. J. Neurosurg. Spine.

[6] Acaroglu E., Guler U.O., Olgun Z.D., Yavuz Y., Pellise F., Domingo-Sabat M., Yakici S., Alanay A., Perez-Grueso F.S., Yavuz Y. (2015). Multiple Regression Analysis of Factors Affecting Health-Related Quality of Life in Adult Spinal Deformity. Spine Deform..

[7] Birknes J.K., White A.P., Albert T.J., Shaffrey C.I., Harrop J.S. (2008). Adult Degenerative Scoliosis: A Review. Neurosurgery.

[8] Daubs M.D., Lenke L.G., Bridwell K.H., Kim Y.J., Hung M., Cheh G., Koester L.A. (2013). Does Correction of Preoperative Coronal Imbalance Make a Difference in Outcomes of Adult Patients with Deformity?. Spine.

[9] Barrey C., Roussouly P., Perrin G., Le Huec J.-C. (2011). Sagittal Balance Disorders in Severe Degenerative Spine. Can We Identify the Compensatory Mechanisms?. Eur. Spine J..

[10] Baghdadi Y.M.K., Larson A.N., Dekutoski M.B., Cui Q., Sebastian A.S., Armitage B.M., Nassr A. (2014). Sagittal Balance and Spinopelvic Parameters after Lateral Lumbar Interbody Fusion for Degenerative Scoliosis: A Case-Control Study. Spine.

[11] Koller H., Pfanz C., Meier O., Hitzl W., Mayer M., Bullmann V., Schulte T.L. (2016). Factors Influencing Radiographic and Clinical Outcomes in Adult Scoliosis Surgery: A Study of 448 European Patients. Eur. Spine J..

[12] Ploumis A., Liu H., Mehbod A.A., Transfeldt E.E., Winter R.B. (2009). A Correlation of Radiographic and Functional Measurements in Adult Degenerative Scoliosis. Spine.

[13] Schwab F.J., Lafage V., Farcy J.-P., Bridwell K.H., Glassman S., Shainline M.R. (2008). Predicting Outcome and Complications in the Surgical Treatment of Adult Scoliosis. Spine.

[14] Bao H., Yan P., Qiu Y., Liu Z., Zhu F. (2016). Coronal Imbalance in Degenerative Lumbar Scoliosis: Prevalence and Influence on Surgical Decision-Making for Spinal Osteotomy. Bone Joint J..

[15] Matsumura A., Namikawa T., Kato M., Hori Y., Hidaka N., Nakamura H. (2020). Factors Related to Postoperative Coronal Imbalance in Adult Lumbar Scoliosis. J. Neurosurg. Spine.

[16] Theologis A.A., Lertudomphonwanit T., Lenke L.G., Bridwell K.H., Gupta M.C. (2021). The Role of the Fractional Lumbosacral Curve in Persistent Coronal Malalignment Following Adult Thoracolumbar Deformity Surgery: A Radiographic Analysis. Spine Deform..

[17] Zhang Z., Song K., Wu B., Chi P., Wang Z., Wang Z. (2019). Coronal Imbalance in Adult Spinal Deformity Following Posterior Spinal Fusion with Instrument: A Related Parameters Analysis. Spine.

[18] Zhang J., Wang Z., Chi P., Chi C. (2020). Orientation of L4 Coronal Tilt Relative to C7 Plumb Line as a Predictor for Postoperative Coronal Imbalance in Patients with Degenerative Lumbar Scoliosis. Sci. Rep..

[19] Campbell P.G., Nunley P.D. (2018). The Challenge of the Lumbosacral Fractional Curve in the Setting of Adult Degenerative Scoliosis. Neurosurg. Clin..

[20] Lewis S.J., Keshen S.G., Kato S., Dear T.E., Gazendam A.M. (2018). Risk Factors for Postoperative Coronal Balance in Adult Spinal Deformity Surgery. Glob. Spine J..

[21] Tanaka N., Ebata S., Oda K., Oba H., Haro H., Ohba T. (2020). Predictors and Clinical Importance of Postoperative Coronal Malalignment After Surgery to Correct Adult Spinal Deformity. Clin. Spine Surg..

[22] Obeid I., Berjano P., Lamartina C., Chopin D., Boissière L., Bourghli A. (2019). Classification of Coronal Imbalance in Adult Scoliosis and Spine Deformity: A Treatment-Oriented Guideline. Eur. Spine J..

[23] Ploumis A., Simpson A.K., Cha T.D., Herzog J.P., Wood K.B. (2015). Coronal Spinal Balance in Adult Spine Deformity Patients with Long Spinal Fusions: A Minimum 2- to 5-Year Follow-up Study. J. Spinal Disord. Tech..

[24] Glassman S.D., Berven S., Bridwell K., Horton W., Dimar J.R. (2005). Correlation of Radiographic Parameters and Clinical Symptoms in Adult Scoliosis. Spine.

